# Estimating ambient air pollutant levels in Suzhou through the SPDE approach with R-INLA

**DOI:** 10.1016/j.ijheh.2021.113766

**Published:** 2021-06

**Authors:** Neil Wright, Katherine Newell, Kin Bong Hubert Lam, Om Kurmi, Zhengming Chen, Christiana Kartsonaki

**Affiliations:** a Clinical Trial Service Unit and Epidemiological Studies Unit, Nuffield Department of Population Health, University of Oxford, Oxford, United Kingdom; b Faculty Research Centre for Intelligent Healthcare, Coventry University, Coventry, United Kingdom; c MRC Population Health Research Unit, University of Oxford, Oxford, United Kingdom

**Keywords:** Integrated nested Laplace approximation, Stochastic partial differential equation, Bayesian approach, Ambient air pollution, Covariate misalignment

## Abstract

Spatio–temporal models of ambient air pollution can be used to predict pollutant levels across a geographical region. These predictions may then be used as estimates of exposure for individuals in analyses of the health effects of air pollution. Integrated nested Laplace approximations is a method for Bayesian inference, and a fast alternative to Markov chain Monte Carlo methods. It also facilitates the SPDE approach to spatial modelling, which has been used for modelling of air pollutant levels, and is available in the R-INLA package for the R statistics software. Covariates such as meteorological variables may be useful predictors in such models, but covariate misalignment must be dealt with. This paper describes a flexible method used to estimate pollutant levels for six pollutants in Suzhou, a city in China with dispersed air pollutant monitors and weather stations. A two-stage approach is used to address misalignment of weather covariate data.

## 1 Introduction

Research into the health effects of ambient air pollution requires long-term measurements of pollutant exposure at the individual level. Studies on longer-term effects of air pollution exposure in China (and elsewhere) have used averaged concentrations from static ambient air pollution monitors (Cao et al., 2011; Chen et al., 2016; Dong et al., 2012; Li et al., 2014; Zhang et al., 2011; Zhou et al., 2014) or satellite data (Peng et al., 2017; Yin et al., 2017) for exposure information. For analyses that require individual exposure levels for study participants, spatio–temporal models of ambient air pollution may be used to predict pollutant levels across a geographical region. Predictions at individuals’ residential or employment locations can then be used as estimates of ambient air pollution exposure.

Bayesian inference offers a practical method for applying such spatio–temporal models and producing predictions. Integrated nested Laplace approximations (INLA) (Rue et al., 2009) allow fast computation for Bayesian inference and enable the use of the SPDE approach for spatial modelling (Lindgren et al., 2011). These methods have been used in modelling of air pollutant levels in Italy (Cameletti et al., 2013; Fioravanti et al., 2021) and England (Blangiardo et al., 2016).

Meteorological variables can be useful predictors in models of ambient air pollution, but weather station locations may not coincide with the pollutant monitor locations or locations where predictions are sought. This is a case of the problem of covariate misalignment, where covariate data are not available at the same locations as observed dependent data. Joint modelling (Barber et al., 2016) or error models can be used to incorporate such covariates while accounting for uncertainty.

We obtained estimated pollutant exposure levels for participants in Suzhou in the China Kadoorie Biobank study. Data were available from static monitors for six pollutants: fine (PM_2.5_), and coarse (PM_10_) particulate matter, sulphur dioxide (SO_2_), nitrogen dioxide (NO_2_), carbon monoxide (CO), and ozone (O_3_). We used Bayesian spatial-temporal models to predict monthly levels of each pollutant at all clinic locations in the area. These predictions can be used as proxies for individual pollution exposure in analyses of health outcomes. This method exploits the spatial information from having monitors in different locations, providing localised exposure estimates that are not available by averaging pollution levels across the study area. Weather data were also available from stations in the city. However, the locations of weather monitors did not coincide with the pollutant monitors. Previous work (Blangiardo et al., 2016; Cameletti et al., 2013; Fioravanti et al., 2021) has used covariates that are fixed over space, or used the geographically closest available measurements. Given the limited number and placement of weather monitors, we used a two-stage approach to address misalignment of weather covariate data, and compare four models for including weather covariates in the pollutant models.

## 2 Background

### 2.1 China Kadoorie Biobank study

The China Kadoorie Biobank study (Chen et al., 2005) recruited 512,726 participants between 2004 and 2008, from ten diverse areas of China. Participants are followed up for a wide range of health outcomes via linkages with health insurance systems, established disease surveillance systems and death registries. Details of the study design and methods have been reported previously (Chen et al, 2005, 2011). In Suzhou, 53,269 study participants were recruited each of whom is linked to one of 77 local clinics. One clinic located outside the urban area of Suzhou was excluded from this analysis.

### 2.2 INLA and SPDE spatial models

Integrated nested Laplace approximations (INLA) (Rue et al., 2009; Wang et al., 2018) is a fast alternative to Markov chain Monte Carlo (MCMC) methods for Bayesian inference from latent Gaussian models. The method uses numerical integration and Laplace approximations for approximate Bayesian inference and is implemented in the R package R-INLA (R-INLA, 2020). The package includes many latent models, including SPDE spatial models (Lindgren et al., 2011), error models (Muff et al., 2013), and auto-regressive models. Posterior predictive distributions produced by fitting Bayesian models can be used to generate point or ranges of predictions.

The SPDE approach to spatial modelling, implemented in the R-INLA package, involves representing a continuously indexed Gaussian field with Matérn covariance as a discretely indexed Gaussian Markov random field (GMRF). This is achieved by means of a basis function representation defined on a triangulation of the domain. The GMRF has a sparse precision matrix and so computationally efficient methods for matrix factorisation, and INLA methods for Bayesian inference, can be used (Bakka et al., 2018; Bivand et al., 2015; Blangiardo et al., 2013; Blangiardo and Cameletti, 2015; Gomez-Rubio, 2020; Krainski et al., 2019; Lindgren et al., 2011; Lindgren and Rue, 2015; Moraga, 2019). The SPDE approach to spatial modelling and the Matérn covariance function and its parameters are well described in Chapter 6 of Blangiardo and Cameletti (2015). Separable space-time models, defined by the Kronecker product between the two precision matrices, can be constructed using the group feature in R-INLA. This allows spatial and temporal correlations to be jointly modelled. This form of spatio–temporal model is well described in Chapter 7 of Blangiardo and Cameletti (2015) and Chapter 10 of Moraga (2019). These methods have previously been used for spatio–temporal modelling of PM_10_ levels in Italy (Cameletti et al., 2013; Fioravanti et al., 2021) and NO_2_ levels in England (Blangiardo et al., 2016).

### 2.3 Pollution and weather data

**Fig. 1:**
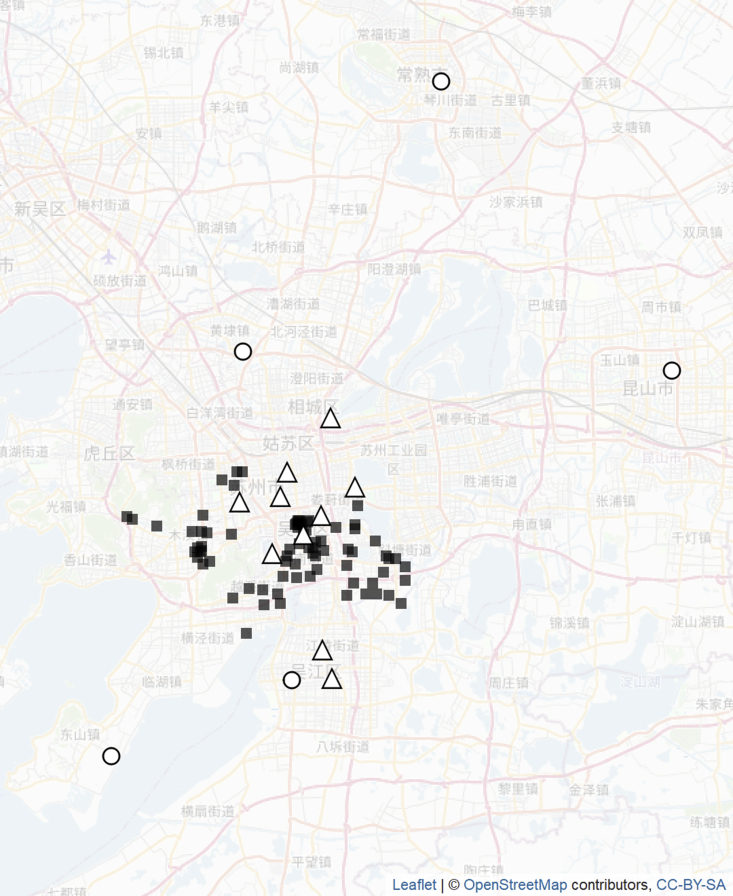
Locations of clinics (black squares), pollutant monitors (triangles) and weather stations (circles).

The data included daily average measurements of six pollutants: particulate matter with diameter of 2.5 μm or less (PM_2.5_), particulate matter with a diameter between 2.5 and 10 μm (PM_10_), sulphur dioxide (SO_2_), nitrogen dioxide (NO_2_), carbon monoxide (CO), and ozone (O_3_). Measurements were available between January 2013 and December 2015 from up to 10 pollution monitors situated in Suzhou (as shown in Fig. 1). Daily weather data, including temperature, pressure, precipitation and wind speed, were available from five monitors in the region from January 2013 to June 2016. The locations of the weather monitors are also shown in Fig. 1 and do not coincide with the locations of the pollution monitors or clinics. Five geographic covariates were available for all locations: elevation; distance to nearest major road; distance to nearest motorway; total length of major roads and motorways in a 1 km radius; and land use (a binary variable representing “urban” or “non-urban”). Elevation values were interpolated from the values of the four nearest raster cells.

## 3 Methods

In order to address the misalignment problem and use weather data variables as covariates in the pollutant models, two stages of models were used. Firstly, models for each weather variable were used to obtain predictions of each weather variable at pollution monitor and clinic locations. These predictions were then included as covariates in the models for the pollutants. Approximate Bayesian inference was performed using INLA with the R-INLA software package (R-INLA, 2020) for R.

This analysis used pollutant and weather variables aggregated to monthly means. This reduced the size of the data being used and number of values to be estimated, and made distributions approximately normal. For example, daily rainfall data are highly skewed with many zeroes, but the observed monthly average rainfall has a symmetric distribution. Observed daily pollutant levels of zero were set to missing, as these were believed to indicate errors in the data.

### 3.1 Meshes for SPDE spatial model

**Fig. 2:**
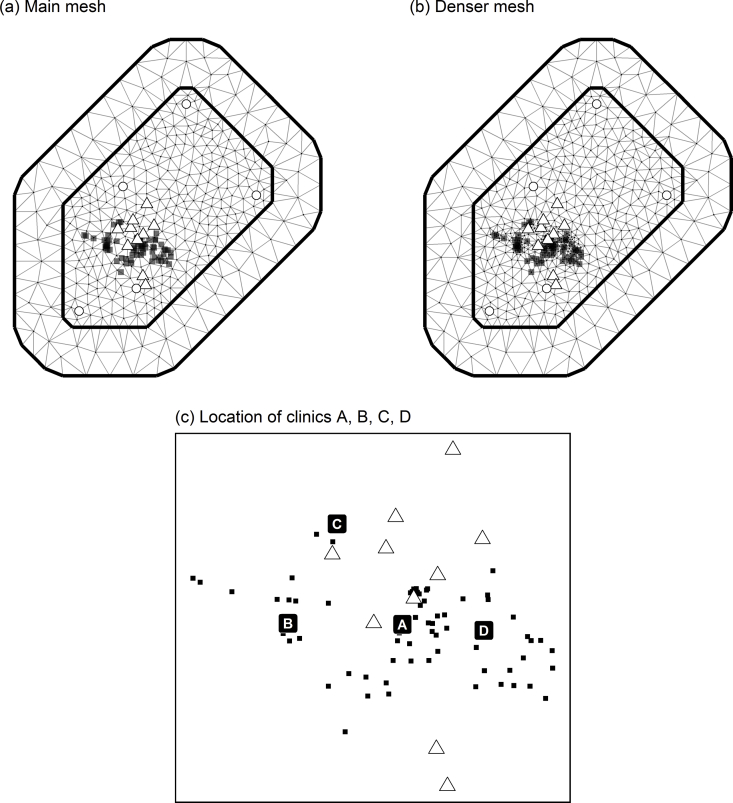
Meshes and locations of clinics (black squares), pollutant monitors (triangles) and weather stations (circles).

A mesh (triangulation) of the region was required to apply SPDE spatial models. The same mesh was used for all weather variable and pollutant models. Latitude and longitude coordinates of all locations were converted to Universal Transverse Mercator (UTM) coordinates. All clinic and monitor locations are in UTM zone 51. These coordinates were then re-scaled with centre equal to the midpoint of all monitor and clinic locations and so that 1 unit equals approximately 1 km. The R-INLA inla.mesh.2d function, which employs constrained refined Delaunay triangulation, was then used to construct a triangular mesh on the region. The domain was formed by the convex hull of all weather station, pollutant monitor and clinic locations, with an inner extension of 5 km and an outer extension of 15 km. The locations of all pollution monitors and weather monitors were used as initial triangulation nodes. The maximum edge ledge was set to 5 km (10 km in the outer extension), the minimum triangle angle was set to 28° (18° in the outer extension), and the minimum distance between points was set to 0.1 km. A denser mesh was also constructed using the locations of all clinics, as well as weather stations and pollutant monitors, as initial triangulation nodes. The weather and pollutant prediction models were additionally fit using this mesh, and point predictions (median of the posterior predictive distribution) of the pollutants were compared between using either mesh by Pearson correlation. The main mesh had 432 nodes and the denser mesh 711 nodes. The meshes are shown in Fig. 2.

### 3.2 Weather models

From the weather data, four variables were selected representing temperature (daily average temperature), humidity (daily average humidity), wind speed (daily 10 min maximum wind speed), and precipitation (total 24 h precipitation). The wind speed variable was log transformed. Each of the four weather variables was aggregated to monthly means and then re-scaled to have a mean of zero and a variance of one.

Each of the four weather variables was modelled as a Gaussian response. Model predictors with and without linear effects for space and time trends were compared using the Watanabe–Akaike (or “Widely Applicable”) information criterion (WAIC) (Gelman et al., 2014; Watanabe, 2010). Calendar month was included as a factor. No level was dropped but the intercept term was dropped, so that prior distributions were exchangeable for levels of this factor. All models included a space-time model, using an SPDE spatial model (i.e. an approximation to a Gaussian field with Matérn covariance) for spatial correlations and a first order auto-regressive model for temporal correlations. Details and formulae for the models are provided in a supplementary file.

### 3.3 Pollutant models

After aggregation to monthly means, pollutant levels were log transformed and then modelled as Gaussian responses. The model predictors included spatial trends, a linear time trend, calendar month as a factor, five geographic covariates, and a space-time model with an SPDE spatial model (i.e. an approximation to a Gaussian field with Matérn covariance) and a first order auto-regressive model for temporal correlations. Continuous covariates were re-scaled to have mean zero and variance one for the pollution monitor and clinic locations. Spatial trends were included using terms for the x- and y-coordinates and the square of x- and y-coordinates. Allowing for a quadratic shape of trends (on the log scale for pollutants) prevented simple linear trends from being extrapolated in predictions for clinic locations far from the centre of the region. In particular, including only a simple linear trend led to extreme, implausible predictions for SO_2_ levels at clinic locations in the far West of the region.

Four different approaches to include the standardised weather covariates in the pollutant models were compared:

1. Exclude weather covariates from the model predictor.
2. Include the mean of the values from each of the weather monitors, so that the same value is used for every location at the same time point.
3. Use the mean of the posterior predictive distribution at each location and time point, for each weather variable.
4. Use an error model. This was a Berkson error model, with observed values equal to the mean of the posterior predictive distribution at each location and time point, and precision fixed and equal to the precision of the posterior predictive distribution. This approach is similar to that investigated by Foster et al. (2012).

There is collinearity between the weather variables and calendar month, which complicates the interpretation of individual coefficients. However, the aim of these models is prediction of pollutant levels, rather than inference for individual coefficients, so this is not a concern and the prediction ability of the models is not affected (Shmueli, 2010).

Models were also fit for each pollutant excluding the SPDE model, but including the temporal first order auto-regressive random effects for stations. This allowed comparison between models which account for spatial correlation and models which ignore spatial correlation.

Details and formulae for the models are provided in a supplementary file.

### 3.4 Prediction models

Given a model with response variable Y and the predictor η,

$$Y\sim Normal\left(\eta ,\sigma_{e}^{2}\right)$$

the posterior distribution of the predictor η is the posterior distribution of the mean response, not the posterior predictive distribution of the response itself. To obtain posterior predictive distributions of the response (including uncertainty due to all sources of error – modelled by $\sigma_{e}^{2}$) an adapted formulation of the model was used:

$$Y\sim Normal\left(\theta ,e^{-20}\right)$$

The precision of the Gaussian response was fixed to be very large ($e^{20}$) so that the response Y is (effectively) equal to the value of the predictor θ. An independent and Gaussian distributed random effect was then added to the predictor, so θ=η+ε and $\varepsilon \sim Normal\left(0,\sigma_{e}^{2}\right)$. This strategy means that the posterior distribution of the predictor θ is the posterior predictive distribution of the response.

Posterior predictive distributions for pollutants were summarised by medians and 95% equal tailed intervals. Examples of the R code used for prediction models, for humidity and PM_10_, are provided in a supplementary file.

### 3.5 Priors

The prior distributions for calendar month and other fixed effect parameters were Normal with mean 0 and precision 0.001. The priors for the precision of the responses were Gamma with a shape parameter of 1 and an inverse scale parameter of $5\times 10^{-5}$. The priors for the coefficient, a, of the first order auto-regressive model were given by $log(\left(1+a\right)/\left(1-a\right))\sim Normal\left(0,1{/}_{0.15}\right)$. Normal priors were used for the coefficients of Berkson error models with mean 1 and precision 0.001. The mean and precision parameters for the error models were fixed values and therefore do not have prior or posterior distributions.

For the SPDE spatial models, penalised complexity (PC) priors were used with P(r<10)=0.5 and P(σ>1)=0.5, where r is the range and σ the standard deviation of the field.

### 3.6 Posteriors and model fit statistics

Posterior distributions for parameters and hyperparameters were transformed to the original scale of the dependent variable as applicable, and then summarised by medians and 95% highest posterior density (HPD) intervals. As pollutant levels were log transformed in the models, the exponentiated covariate coefficients are interpretable as ratios. For the SPDE models, hyperparameters were transformed to the range and variance.

Models were compared using the Watanabe–Akaike (or “Widely Applicable”) Information Criterion (WAIC), a Bayesian approach for estimating out-of-sample prediction error (Gelman et al., 2014; Watanabe, 2010).

To further asses the performance of the modelling approach, pollutant prediction models were also applied after excluding a sample of 50 pollutant observations. The sample was a simple random sample from all 348 combinations of monitor location and month (in which observed data were available). The RMSE and Pearson correlation between predicted (median of posterior predictive distribution) and observed values were then calculated.

## 4 Results

Observed weather and pollutant data are summarised in Table 1, using daily values and monthly means calculated for each monitor. Precipitation data are missing for 458 daily observations. There are at least 10,209 observations for each pollutant across the three year period.

**Table 1:** Summaries of observed data from five weather monitors (from January 2013 to June 2016) and up to 13 pollutant monitors (from January 2013 to December 2015).

	N	Mean	SD	Minimum	Median	Maximum
**Daily values**						
Weather variables						
Temperature (°C)	6385	16.8	8.8	−6.1	17.7	36.2
Wind speed (m/s)	6385	4.6	1.5	1.3	4.4	15.7
Humidity (%)	6385	73.8	13.5	29.0	75.0	100.0
Precipitation (mm)	5927	4.0	11.2	0.0	0.0	170.2
Pollutants (μg/m³)						
PM_10_	10,230	87.3	48.5	3.0	76.0	429.0
PM_2.5_	10,209	63.1	39.1	3.0	55.0	405.0
SO_2_	10,225	24.2	15.3	1.0	21.0	164.0
CO (mg/m³)	10,237	0.9	0.4	0.1	0.8	3.5
NO_2_	10,240	50.3	22.1	5.0	47.0	321.0
O_3_	10,213	97.4	52.0	1.0	90.0	1251.0
**Monthly values**						
Weather variables						
Temperature (°C)	210	16.8	8.3	3.6	16.8	32.3
Wind speed (m/s)	210	4.6	0.7	3.5	4.6	6.7
Humidity (%)	210	73.8	6.7	56.4	74.0	91.9
Precipitation (mm)	210	4.1	3.5	0.2	3.3	25.0
Pollutants (μg/m³)						
PM_10_	348	87.2	27.2	43.2	80.2	194.3
PM_2.5_	348	63.0	23.5	24.5	58.7	155.6
SO_2_	348	24.1	10.2	8.5	21.9	63.4
CO (mg/m³)	348	0.9	0.2	0.4	0.9	1.8
NO_2_	348	50.7	15.1	24.3	48.5	109.3
O_3_	348	95.9	34.5	18.6	104.5	182.2

### 4.1 Weather models

Including linear trends for time or space did not consistently decrease the WAIC values, so trends were not included in the weather prediction models. Medians and 95% HPD intervals of posterior distributions for the calendar month intercepts and hyperparameters of the four weather models are given in Table 2. Seasonal patterns are present for each of the weather variables. In particular, temperature and precipitation have much higher intercepts during the summer months (June to September) as in the observed data. The ranges of the SPDE models are large (posterior medians from 108 km to 379 km). Temperature, wind speed and humidity have high auto-correlation between months (posterior median of the AR coefficients of 0.96 or greater), whereas precipitation has weak auto-correlation (posterior median of the AR coefficient is 0.18).

**Table 2:** Medians and 95% HPD intervals of posterior distributions from models of monthly weather variables.

	Temperature (°C)	log (Wind speed m/s)	Humidity (%)	Precipitation (mm)
**Month intercepts**				
January	4.97 (−3.90, 13.72)	1.42 (0.64, 2.20)	73.93 (33.57, 114.84)	1.57 (−0.91, 4.06)
February	6.29 (−2.60, 15.04)	1.50 (0.72, 2.29)	76.31 (35.89, 117.27)	3.49 (1.01, 5.97)
March	11.01 (2.11, 19.77)	1.53 (0.75, 2.32)	71.92 (31.48, 112.91)	2.69 (0.21, 5.18)
April	16.11 (7.21, 24.87)	1.59 (0.81, 2.37)	70.57 (30.14, 111.57)	4.54 (2.05, 7.02)
May	21.14 (12.25, 29.89)	1.54 (0.75, 2.32)	73.82 (33.41, 114.80)	4.33 (1.85, 6.82)
June	24.01 (15.13, 32.74)	1.45 (0.66, 2.23)	83.38 (43.03, 124.30)	10.81 (8.33, 13.29)
July	28.53 (19.60, 37.31)	1.53 (0.75, 2.31)	77.39 (36.80, 118.57)	4.93 (2.08, 7.79)
August	28.15 (19.19, 36.97)	1.50 (0.71, 2.28)	78.71 (37.95, 120.05)	4.78 (1.91, 7.65)
September	24.09 (15.11, 32.92)	1.46 (0.67, 2.24)	78.91 (38.07, 120.33)	3.39 (0.52, 6.25)
October	19.20 (10.23, 28.03)	1.41 (0.62, 2.19)	74.32 (33.48, 115.75)	4.16 (1.30, 7.03)
November	13.20 (4.24, 22.02)	1.38 (0.60, 2.16)	77.13 (36.37, 118.47)	2.30 (−0.57, 5.17)
December	5.82 (−3.10, 14.61)	1.41 (0.63, 2.20)	70.13 (29.52, 111.29)	1.28 (−1.58, 4.13)
**Hyperparameters**				
SD for the Gaussian observations	0.08 (0.06, 0.09)	0.04 (0.03, 0.04)	1.15 (0.95, 1.38)	0.03 (0.01, 0.08)
Range of SPDE model (km)	378.63 (308.76, 461.48)	129.85 (99.81, 163.70)	247.64 (193.90, 310.69)	108.06 (91.61, 126.83)
Variance of SPDE model	0.01 (0.00, 0.03)	2.61 (0.81, 6.18)	1.04 (0.35, 2.44)	0.20 (0.16, 0.25)
Coefficient of AR model	0.96 (0.92, 0.99)	0.99 (0.97, 1.00)	0.96 (0.92, 0.99)	0.18 (0.03, 0.33)

Predicted temperature values (medians of posterior predictive distributions) at pollutant monitor and clinic locations vary between 4.02 and 32.18 °C. Wind speed predictions range from 3.38 to 5.83 m/s, and humidity from 56.84 to 84.97%. Predicted precipitation values range from 0.15 to 16.97 mm.

### 4.2 Pollutant models

WAIC values for five models for each pollutant are given in Table 3. The models including error models for weather variables have lower WAIC values - indicating a better fit to the data - than models which use other methods to incorporate weather covariates, for all pollutants. For all pollutants, except PM_10_, the WAIC for models excluding an SPDE spatial model is larger indicating that accounting for spatial correlations with an SPDE model improves the fit of the models. This also allows for individual predictions of pollutant levels at locations across the region. The following results and predictions use models which include error models for the weather covariates.

**Table 3:** WAIC values for monthly pollutant models with different methods for using weather covariates.

Model	PM_10_	PM_2.5_	SO_2_	CO	NO_2_	O_3_
1. Exclude weather covariates	−810.49	−932.64	−328.12	−467.15	−856.00	−1026.77
2. Mean values	−838.14	−1001.50	−350.41	−468.53	−2044.77	−2051.05
3. Means of posterior predictive distribution	−833.04	−998.91	−341.33	−467.04	−2027.96	−2057.84
**4. Error models**	**−1302.55**	**−1046.17**	**−389.43**	**−646.71**	**−2097.08**	**−2086.67**
5. Excluding SPDE model	−1885.11	−723.74	−319.38	−472.62	−1078.37	−2084.94
6. Excluding quadratic spatial terms	−2053.49	−1009.52	−378.29	−690.40	−2067.99	−2139.77

Medians and 95% HPD intervals of posterior distributions for the parameters and hyperparameters of the six pollutant models are given in Table 4, Table 5. Collinearity between calendar month intercepts and weather variables inhibits clear interpretation of these parameters. The particulate matter pollutants have the largest range for the spatial model (posterior medians 42 km and 45 km), followed by CO and SO_2_ (30 km and 14 km), and then NO_2_ and O_3_ (8 km and 6 km).

**Table 4:** Medians and 95% HPD intervals of posterior distributions from models of monthly means of particulate matter pollutants.

	PM_10_	PM_2.5_
**Month intercepts (μg/m³)**		
January	65.69 (26.16, 136.98)	60.28 (20.52, 132.96)
February	52.57 (21.20, 106.33)	44.84 (15.98, 95.07)
March	63.38 (27.48, 116.50)	57.37 (23.88, 109.10)
April	66.83 (30.21, 116.49)	69.61 (31.51, 123.48)
May	83.60 (36.64, 145.90)	93.86 (40.65, 167.06)
June	88.18 (35.63, 158.04)	106.64 (40.72, 199.27)
July	90.04 (31.87, 168.05)	107.34 (33.93, 218.22)
August	91.20 (32.57, 170.06)	107.37 (34.28, 217.05)
September	75.56 (30.68, 135.92)	86.99 (33.60, 162.26)
October	79.34 (35.45, 138.56)	82.90 (36.95, 146.81)
November	77.35 (33.95, 139.55)	75.74 (32.37, 140.85)
December	66.15 (25.18, 142.37)	61.59 (19.71, 142.18)
**Covariate coefficients (ratios)**		
Time trend (per month)	1.00 (0.98, 1.02)	0.99 (0.97, 1.01)
Longitudinal trend (linear term)	1.13 (0.62, 1.76)	0.96 (0.51, 1.56)
Latitudinal trend (linear term)	1.00 (0.78, 1.25)	1.03 (0.79, 1.30)
Longitudinal trend (quadratic term)	1.55 (0.94, 2.25)	1.58 (0.93, 2.33)
Latitudinal trend (quadratic term)	1.07 (0.86, 1.31)	0.87 (0.69, 1.08)
Urban	0.85 (0.74, 0.98)	0.92 (0.80, 1.06)
Elevation (per 10m)	0.96 (0.73, 1.21)	0.85 (0.65, 1.10)
Distance from road (per 0.01)	0.77 (0.40, 1.28)	0.53 (0.27, 0.90)
Distance from motorway (per 0.01)	1.12 (0.98, 1.26)	1.02 (0.89, 1.16)
Length of roads and motorways in vicinity (per 1 km)	1.01 (0.98, 1.04)	1.00 (0.97, 1.03)
**Coefficients in error models (ratios)**		
Temperature (per 10C)	0.71 (0.50, 0.90)	0.59 (0.31, 0.90)
Wind speed (per 1 SD of log (wind speed))	0.94 (0.91, 0.97)	0.97 (0.94, 1.00)
Humidity (per 5%)	0.89 (0.87, 0.91)	0.97 (0.92, 1.01)
Precipitation (per 10 mm)	1.02 (0.90, 1.17)	0.97 (0.79, 1.16)
**Hyperparameters**		
SD of Gaussian observations (on log scale)	0.03 (0.02, 0.04)	0.04 (0.03, 0.05)
Range of the SPDE model (km)	42.36 (31.64, 51.60)	44.90 (28.53, 62.64)
SD of the SPDE model (on log scale)	0.21 (0.17, 0.25)	0.25 (0.18, 0.33)
Coefficient of AR model	0.80 (0.72, 0.86)	0.82 (0.68, 0.91)

**Table 5:** Medians and 95% HPD intervals of posterior distributions from models of monthly means of gaseous pollutants.

	SO_2_	CO	NO_2_	O_3_
**Month intercepts (μg/m³; mg/m³ for CO)**				
January	26.26 (9.79, 52.99)	0.55 (0.01, 2.53)	64.50 (27.95, 117.11)	114.11 (57.02, 192.03)
February	18.24 (7.10, 36.05)	0.47 (0.01, 2.19)	47.24 (20.73, 85.19)	170.10 (86.03, 284.64)
March	25.36 (11.51, 45.60)	0.52 (0.01, 2.37)	66.30 (30.07, 117.38)	150.07 (79.16, 244.05)
April	32.02 (15.61, 54.86)	0.57 (0.02, 2.58)	67.36 (31.03, 117.93)	142.34 (76.50, 228.09)
May	35.70 (16.78, 61.54)	0.57 (0.02, 2.57)	58.56 (26.75, 102.62)	114.50 (60.87, 183.91)
June	42.10 (17.17, 76.31)	0.52 (0.01, 2.36)	56.44 (25.04, 100.51)	97.39 (49.84, 159.54)
July	52.03 (18.22, 101.37)	0.57 (0.01, 2.65)	53.89 (23.22, 98.06)	72.10 (35.11, 120.89)
August	51.60 (18.18, 100.35)	0.60 (0.02, 2.78)	54.95 (23.71, 99.87)	74.32 (36.25, 124.55)
September	45.19 (18.88, 82.12)	0.55 (0.01, 2.49)	56.42 (25.17, 100.18)	89.18 (45.84, 145.70)
October	39.89 (18.88, 68.25)	0.49 (0.01, 2.17)	62.15 (28.45, 108.73)	107.33 (57.24, 172.34)
November	40.12 (18.99, 69.65)	0.58 (0.02, 2.60)	67.05 (30.65, 117.96)	88.62 (47.34, 142.90)
December	31.55 (11.28, 64.90)	0.62 (0.02, 2.90)	66.10 (28.27, 120.83)	95.85 (47.34, 162.26)
**Covariate coefficients (ratios)**				
Time trend (per month)	0.98 (0.97, 0.99)	0.99 (0.97, 1.02)	1.00 (1.00, 1.01)	1.00 (1.00, 1.00)
Longitudinal trend (linear term)	0.42 (0.17, 0.78)	2.19 (0.03, 10.60)	0.71 (0.23, 1.46)	0.81 (0.34, 1.50)
Latitudinal trend (linear term)	1.02 (0.72, 1.38)	1.48 (0.35, 2.92)	0.96 (0.63, 1.36)	0.92 (0.65, 1.23)
Longitudinal trend (quadratic term)	0.85 (0.39, 1.49)	0.72 (0.02, 2.75)	1.61 (0.60, 3.16)	0.48 (0.21, 0.84)
Latitudinal trend (quadratic term)	1.15 (0.84, 1.51)	1.31 (0.33, 2.55)	0.77 (0.53, 1.04)	1.06 (0.79, 1.37)
Urban	1.53 (1.22, 1.88)	1.14 (0.57, 1.78)	0.85 (0.63, 1.09)	1.13 (0.88, 1.40)
Elevation (per 10m)	1.01 (0.67, 1.42)	1.16 (0.21, 2.43)	0.66 (0.40, 0.98)	1.19 (0.78, 1.68)
Distance from road (per 0.01)	1.33 (0.42, 2.80)	1.83 (0.01, 9.25)	0.23 (0.05, 0.58)	2.89 (0.83, 6.43)
Distance from motorway (per 0.01)	0.85 (0.70, 1.01)	1.18 (0.60, 1.83)	0.96 (0.76, 1.18)	0.91 (0.75, 1.09)
Length of roads and motorways in vicinity (per 1 km)	1.00 (0.96, 1.04)	0.98 (0.88, 1.09)	1.02 (0.98, 1.07)	1.01 (0.97, 1.05)
**Coefficients in error models (ratios)**				
Temperature (per 10C)	0.60 (0.35, 0.90)	0.90 (0.69, 1.15)	0.82 (0.63, 1.01)	1.96 (1.54, 2.45)
Wind speed (per 1 SD of log (wind speed))	0.99 (0.90, 1.08)	0.88 (0.84, 0.91)	0.90 (0.88, 0.93)	0.95 (0.89, 0.99)
Humidity (per 5%)	0.90 (0.85, 0.95)	0.97 (0.94, 0.99)	0.95 (0.92, 0.97)	0.97 (0.94, 0.99)
Precipitation (per 10 mm)	1.00 (0.78, 1.26)	1.11 (0.99, 1.24)	1.03 (0.95, 1.12)	0.90 (0.80, 1.00)
**Hyperparameters**				
SD of Gaussian observations (on log scale)	0.10 (0.07, 0.13)	0.07 (0.06, 0.09)	0.01 (0.00, 0.02)	0.01 (0.00, 0.03)
Range of the SPDE model (km)	14.48 (8.04, 22.75)	29.70 (18.49, 46.59)	7.61 (4.26, 11.05)	5.61 (3.66, 7.35)
SD of the SPDE model (on log scale)	0.20 (0.16, 0.25)	0.41 (0.19, 0.94)	0.17 (0.12, 0.21)	0.15 (0.13, 0.17)
Coefficient of AR model	0.60 (0.41, 0.75)	0.96 (0.88, 1.00)	0.79 (0.66, 0.87)	0.59 (0.49, 0.70)

**Fig. 3:**
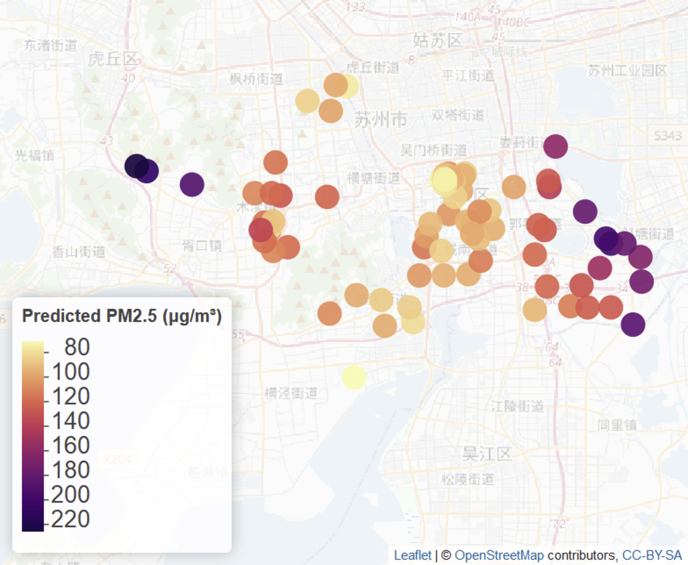
Predicted levels (posterior medians) of PM_2.5_ at clinic locations for January 2014.

**Fig. 4:**
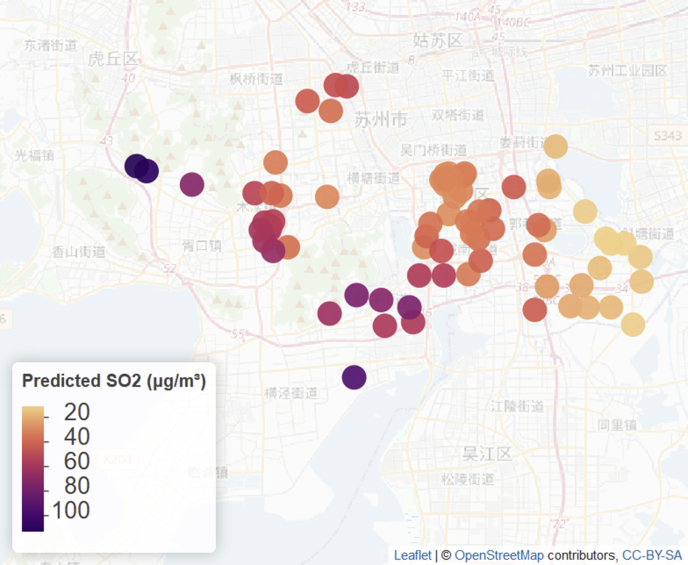
Predicted levels (posterior medians) of SO_2_ at clinic locations for January 2014.

**Fig. 5:**
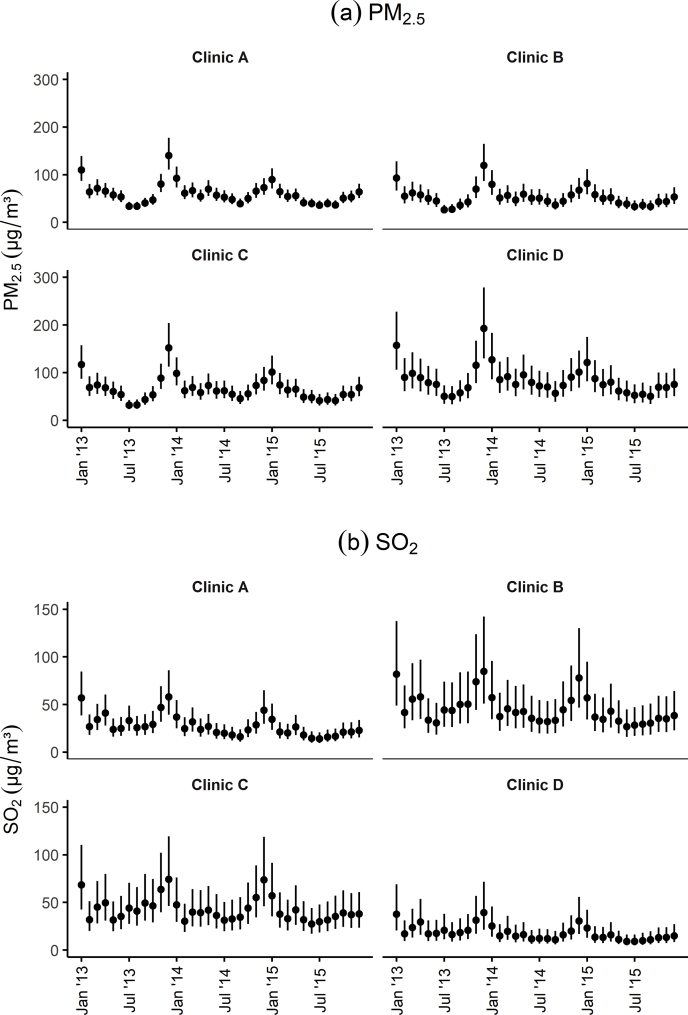
Posterior medians and 95% predictive intervals for pollutant levels at four clinics.

Predicted levels (posterior medians) of PM_2.5_ and SO_2_ for January 2014 at all clinic locations are shown in Fig. 3, Fig. 4. Posterior medians and 95% predictive intervals for PM_2.5_ and SO_2_ at four selected clinic locations (shown in Fig. 2) are given in Fig. 5. Predicted values (medians of posterior predictive distributions) at clinic locations have medians (inter-quartile range) of 66.12 (51.39–88.08) μg/m³ for PM_2.5_, 84.88 (58.65–102.24) μg/m³ for PM_10_, 25.90 (17.13–38.59) μg/m³ for SO_2_, 59.21 (42.65–81.67) μg/m³ for NO_2_, 0.61 (0.42–0.77) mg/m³ for CO, and 91.09 (54.95–134.28) μg/m³ for O_3_.

**Fig. 6:**
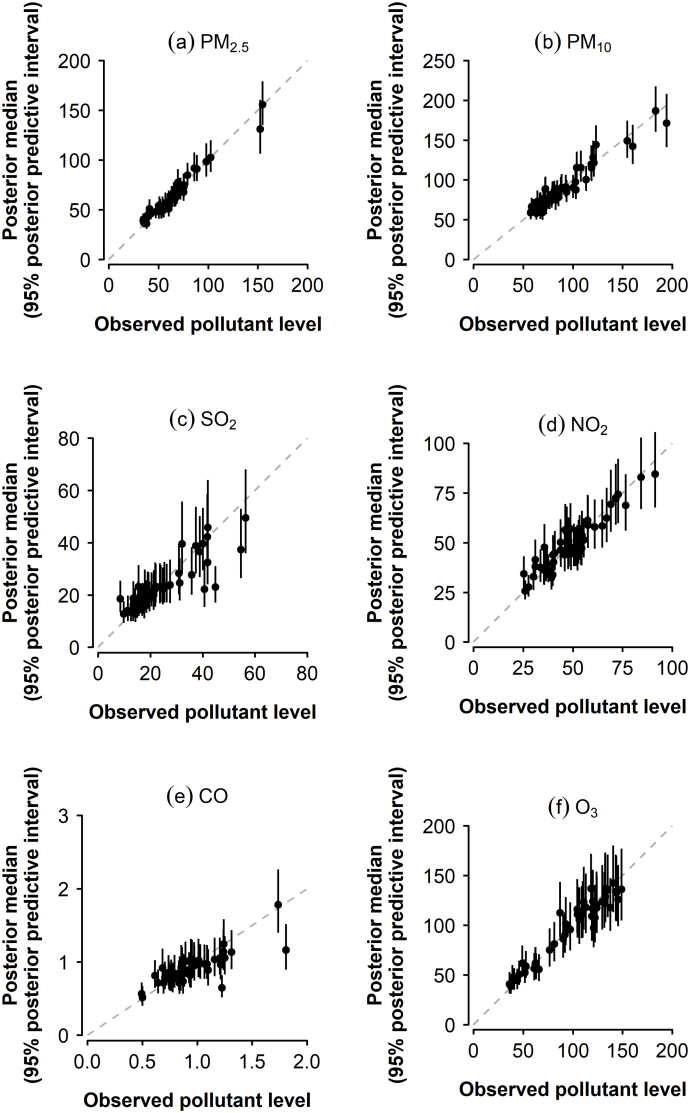
Posterior medians and 95% predictive intervals for pollutant levels for random sample (having excluded observed data when fitting the model.

After fitting the pollutant prediction models while excluding a random sample of fifty observations, posterior medians and 95% predictive intervals for pollutant levels are shown in Fig. 6. RMSE and correlations between predicted values (posterior medians) and observed values are given in Table 6. Correlations range from 0.80 for CO to 0.98 for PM_2.5_.

**Table 6:** RMSE and correlations between predicted values (posterior medians) and observed values for a random sample of fifty observations (excluded when fitting the models).

	PM_10_	PM_2.5_	SO_2_	CO	NO_2_	O_3_
RMSE	8.20	5.03	5.90	0.16	5.05	9.14
Correlation	0.96	0.98	0.87	0.80	0.94	0.97

**Fig. 7:**
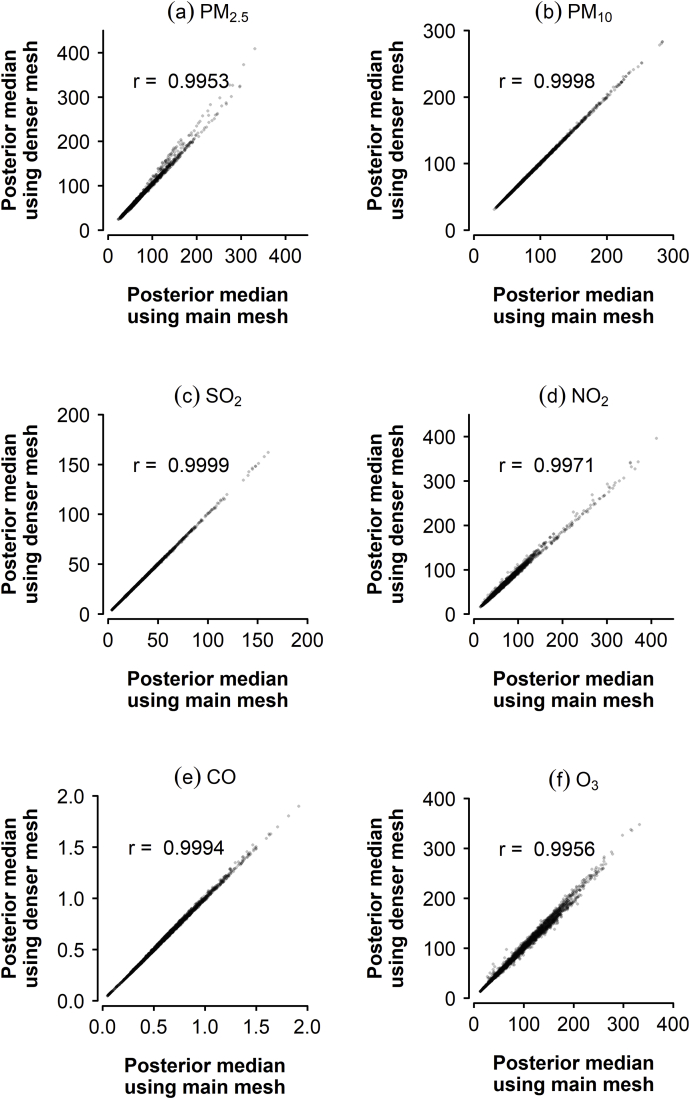
Posterior medians for pollutant levels after using the main mesh and the denser mesh, and Pearson correlation coefficients.

Predicted pollutant levels are very similar (Pearson correlation coefficients greater than 0.99) when using the denser mesh for both weather variable and pollutant models. Posterior medians for pollutant levels after using either mesh are shown in Fig. 7.

## 5 Discussion

We have used Bayesian spatio–temporal models to predict levels of six pollutants at clinic locations in Suzhou, China. Inference was performed using the approximate INLA method and spatial models used the SPDE approach. The application of the SPDE approach for modelling pollutant levels has previously been reported by Cameletti et al. (2013) and Blangiardo et al. (2016). These analyses used covariates measured at or aligned to the same locations as the observed pollutant measurements. We extended this approach using a two-stage method to address misalignment of covariates. After using spatio–temporal models to produce predictions for four meteorological variables at all relevant locations, we used error models to add these as predictors in the models for pollutants. This ensured that the pollutant models incorporated the uncertainty in the predicted weather covariate values. To obtain predictions for pollutant levels at the set of clinic locations we extended the pollutant models so that posterior predictive distributions were obtained directly from R-INLA function calls.

The models and methods described in this paper provide a flexible approach to modelling ambient air pollutant levels in a region with dispersed monitors. The analysis incorporates fixed and time-varying covariate data from several sources, including misaligned covariates for which error models were used to ensure appropriate error propagation. This approach could be adapted for other scenarios, and models can be expanded with comparative ease.

These results are based on monthly pollutant levels, which were aggregated from daily data to monthly means before developing prediction models. However, the models could be adapted to use daily average values for meteorological variables and pollutant levels to enable more detailed time-series analyses. To capture dependencies over time, splines could be used with auto-regressive models for the values at knot locations.

We suggest pollutant levels at clinic locations could be used as proxies for individual exposure. It would be desirable to have individual participant residence, employment and other common locations to estimate exposure, however only clinic location (anticipated to be close to residence) is available in the given data. The methods described could be used to predict pollutant levels at any locations in the city, and if more extensive location data were available more specific estimates of exposure could be calculated.

The limited number of pollutant and weather monitors did not allow for detailed modelling of pollutants and weather variables across the city. It would be preferable to have data from more monitors throughout the city to allow better predictions of levels across the city. Given the available data, we have leveraged the geographic information available to predict pollutant levels at each clinic location. This is an alternative to ignoring the locations of pollutant monitors by using city-wide means in time-series analyses of health outcomes, or using pollutant levels are the nearest monitor as estimated levels at a clinic location.

The ranges of the SPDE models in the weather models are much larger than the extent of the area over which the models were applied. In such cases the model is usually indistinguishable from intrinsic random fields (Lindgren and Rue, 2015), but we do not expect that this affects the utility of predicted weather variables as covariates in the pollutant models.

The narrow locations (East to West) of the pollutant monitors caused a problem with including overall spatial trends. Extrapolating simple linear trends to out-of-sample x-coordinates caused predictions to be implausibly high (with small precision) in some models, but this was tempered by including quadratic terms for spatial trends. Ideally, observed pollutant data would be more geographically diverse. Alternatively, there may be better methods for ensuring reasonable out-of-sample predictions, and this potential problem should be considered when planning this type of analysis.

As an alternative to the error models used here misaligned covariates could be jointly modelled with the pollutant variables of interest. Further, health outcome data could be jointly modelled with pollutant levels. This would allow a single modelling framework for exposure, covariate, and outcome data, at the cost of more complex models and the time and resources for computation. However, the use of INLA as an efficient alternative to MCMC methods could make such an approach feasible.

## Declaration of competing interest

None.
