# Supplementary material for: Estimating ambient air pollutant levels in Suzhou through the SPDE approach with R-INLA
Source: Int J Hyg Environ Health. 2021 Jun;235:113766. doi: 10.1016/j.ijheh.2021.113766 (PMC8223501; doi:10.1016/j.ijheh.2021.113766)
Supplement: Multimedia component 1 [file mmc1.docx]

# Example R code for prediction models

# Humidity prediction model ----------------------------------------------------

# Definition of the SPDE model object
n_months <- as.integer(max(monthly_weatherdata$month))

spde <- inla.spde2.pcmatern(mesh,
 prior.range = c(10, 0.5),
 prior.sigma = c(1, 0.5))

A.est <- inla.spde.make.A(mesh,
 loc = as.matrix(monthly_weatherdata[,c("coord_x_std", "coord_y_std")]),
 group = monthly_weatherdata$month,
 n.group = n_months)

A.pred <- inla.spde.make.A(mesh,
 loc = as.matrix(monthly_pred_weather[,c("coord_x_std", "coord_y_std")]),
 group = monthly_pred_weather$month,
 n.group = n_months)

field.indices <- inla.spde.make.index("field",
 n.spde = mesh$n,
 n.group = n_months)

# Formula
formula <- as.formula("y ~ -1 + ymonth +
 f(field,
 model = spde,
 group = field.group,
 control.group = list(model = 'ar1'))")


# Create stacks
### at weather station locations
stack_est <- inla.stack(data = list(y = monthly_weatherdata[["fitted_humidity_std_mean"]]),
 A = list(A.est,1),
 effects = list(field.indices,
 monthly_weatherdata[, c("ymonth",
 "j")]),
 tag = "est")
### at weather, monitor and clinic locations
stack_pred <- inla.stack(data = list(y = NA),
 A = list(A.pred, 1),
 effects = list(field.indices,
 monthly_pred_weather[, c("ymonth",
 "j")]),
 tag = "pred")


# Run INLA on weather station locations
result_est <- inla(update(formula, ~ . + f(j, model='iid')),
 data = inla.stack.data(stack_est, spde = spde),
 family = "gaussian",
 control.family = list(hyper = list(prec = list(initial = 20, fixed = TRUE))),
 control.predictor = list(A = inla.stack.A(stack_est),
 link = 1,
 compute = FALSE),
 control.fixed = list(expand.factor.strategy = 'inla'),
 verbose = FALSE,
 num.threads = 2)

# Run INLA on weather, monitor and clinic locations
result_pred <- inla(update(formula, ~ . + f(j, model='iid')),
 data = inla.stack.data(stack, spde = spde),
 family = "gaussian",
 control.family = list(hyper = list(prec = list(initial = 20, fixed = TRUE))),
 control.predictor = list(A = inla.stack.A(stack),
 link = 1,
 compute = TRUE),
 control.fixed = list(expand.factor.strategy = 'inla'),
 control.mode = list(theta = result_est$mode$theta,
 restart = FALSE),
 verbose = FALSE,
 num.threads = 2)


# extract predictions
indexes_pred <- inla.stack.index(stack,"pred")$data

monthly_pred_weather[["fitted_humidity_std_mean"]] <-
 results_pred$summary.fitted.values[indexes_pred,"mean"]
monthly_pred_weather[["fitted_humidity_std_prec"]] <-
 1/(results_pred$summary.fitted.values[indexes_pred,"sd"]^2)


# PM10 prediction model ----------------------------------------------------

# Add quadratic coordinate columns --------------------------------------------
monthly_monitordata$coord_x_std_2 <- monthly_monitordata$coord_x_std^2
monthly_monitordata$coord_y_std_2 <- monthly_monitordata$coord_y_std^2

monthly_pred_pollutant$coord_x_std_2 <- monthly_pred_pollutant$coord_x_std^2
monthly_pred_pollutant$coord_y_std_2 <- monthly_pred_pollutant$coord_y_std^2


# Join predicted weather data to pollutant data
monthly_monitordata <- monthly_pred_weather %>%
 select(month, station, starts_with("fitted")) %>%
 right_join(monthly_monitordata, by = c("station","month"))

monthly_pred_pollutant <- monthly_pred_weather %>%
 select(month, loc, starts_with("fitted")) %>%
 right_join(monthly_pred_pollutant, by = c("loc","month"))


# Definition of the SPDE model object
n_months <- as.integer(max(monthly_monitordata$month))

spde <- inla.spde2.pcmatern(mesh,
 prior.range = c(10, .5),
 prior.sigma = c(1, .5))

A.est <- inla.spde.make.A(mesh,
 loc = as.matrix(monthly_monitordata[,c("coord_x_std","coord_y_std")]),
 group = monthly_monitordata$month,
 n.group = n_months)

A.pred <- inla.spde.make.A(mesh,
 loc = as.matrix(monthly_pred_pollutant[,c("coord_x_std","coord_y_std")]),
 group = monthly_pred_pollutant$month,
 n.group = n_months)

field.indices <- inla.spde.make.index("field",
 n.spde = mesh$n,
 n.group = n_months)


# Formulae
formula <- list()
formula[["a"]] <- as.formula("y ~ -1 + coord_x_std + coord_y_std +
 coord_x_std_2 + coord_y_std_2 +
 ymonth + month +
 elevation_std +
 distroad_std + distmway_std + lengthroad_std +
 urban +
 f(fitted_temp_std_mean,
 model = 'meb',
 scale = c(monthly_monitordata[['fitted_temp_std_prec']]),
 values = c(monthly_monitordata[['fitted_temp_std_mean']]),
 hyper = list(prec = list(initial = log(1), fixed = TRUE))) +
 f(fitted_windspeed_std_mean,
 model = 'meb',
 scale = c(monthly_monitordata[['fitted_windspeed_std_prec']]),
 values = c(monthly_monitordata[['fitted_windspeed_std_mean']]),
 hyper = list(prec = list(initial = log(1), fixed = TRUE))) +
 f(fitted_humidity_std_mean,
 model = 'meb',
 scale = c(monthly_monitordata[['fitted_humidity_std_prec']]),
 values = c(monthly_monitordata[['fitted_humidity_std_mean']]),
 hyper = list(prec = list(initial = log(1), fixed = TRUE))) +
 f(fitted_rainfall_std_mean,
 model = 'meb',
 scale = c(monthly_monitordata[['fitted_rainfall_std_prec']]),
 values = c(monthly_monitordata[['fitted_rainfall_std_mean']]),
 hyper = list(prec = list(initial = log(1), fixed = TRUE))) +
 f(field,
 model = spde,
 group = field.group,
 control.group = list(model = 'ar1'))")

formula[["b"]] <- as.formula("y ~ -1 + coord_x_std + coord_y_std +
 coord_x_std_2 + coord_y_std_2 +
 ymonth + month +
 elevation_std +
 distroad_std + distmway_std + lengthroad_std +
 urban +
 f(fitted_temp_std_mean,
 model = 'meb',
 scale = c(monthly_monitordata[['fitted_temp_std_prec']],
 monthly_pred_pollutant[['fitted_temp_std_prec']]),
 values = c(monthly_monitordata[['fitted_temp_std_mean']],
 monthly_pred_pollutant[['fitted_temp_std_mean']]),
 hyper = list(prec = list(initial = log(1), fixed = TRUE))) +
 f(fitted_windspeed_std_mean,
 model = 'meb',
 scale = c(monthly_monitordata[['fitted_windspeed_std_prec']],
 monthly_pred_pollutant[['fitted_windspeed_std_prec']]),
 values = c(monthly_monitordata[['fitted_windspeed_std_mean']],
 monthly_pred_pollutant[['fitted_windspeed_std_mean']]),
 hyper = list(prec = list(initial = log(1), fixed = TRUE))) +
 f(fitted_humidity_std_mean,
 model = 'meb',
 scale = c(monthly_monitordata[['fitted_humidity_std_prec']],
 monthly_pred_pollutant[['fitted_humidity_std_prec']]),
 values = c(monthly_monitordata[['fitted_humidity_std_mean']],
 monthly_pred_pollutant[['fitted_humidity_std_mean']]),
 hyper = list(prec = list(initial = log(1), fixed = TRUE))) +
 f(fitted_rainfall_std_mean,
 model = 'meb',
 scale = c(monthly_monitordata[['fitted_rainfall_std_prec']],
 monthly_pred_pollutant[['fitted_rainfall_std_prec']]),
 values = c(monthly_monitordata[['fitted_rainfall_std_mean']],
 monthly_pred_pollutant[['fitted_rainfall_std_mean']]),
 hyper = list(prec = list(initial = log(1), fixed = TRUE))) +
 f(field,
 model = spde,
 group = field.group,
 control.group = list(model = 'ar1'))")


# Create stacks
### at monitor locations
stack_est <- inla.stack(data = list(y = monthly_monitordata[["pm10_log"]]),
 A = list(A.est, 1),
 effects = list(field.indices,
 monthly_monitordata[, c("coord_x_std",
 "coord_y_std",
 "coord_x_std_2",
 "coord_y_std_2",
 "month",
 "ymonth",
 "elevation_std",
 "distroad_std",
 "distmway_std",
 "lengthroad_std",
 "urban",
 "fitted_temp_std_mean",
 "fitted_windspeed_std_mean",
 "fitted_humidity_std_mean",
 "fitted_rainfall_std_mean",
 "fitted_temp_std_prec",
 "fitted_windspeed_std_prec",
 "fitted_humidity_std_prec",
 "fitted_rainfall_std_prec",
 "j")]),
 tag = "est")

### at clinic locations
stack_pred <- inla.stack(data = list(y = NA),
 A = list(A.pred, 1),
 effects = list(field.indices,
 monthly_pred_pollutant[, c("coord_x_std",
 "coord_y_std",
 "coord_x_std_2",
 "coord_y_std_2",
 "month",
 "ymonth",
 "elevation_std",
 "distroad_std",
 "distmway_std",
 "lengthroad_std",
 "urban",
 "fitted_temp_std_mean",
 "fitted_windspeed_std_mean",
 "fitted_humidity_std_mean",
 "fitted_rainfall_std_mean",
 "fitted_temp_std_prec",
 "fitted_windspeed_std_prec",
 "fitted_humidity_std_prec",
 "fitted_rainfall_std_prec",
 "j")]),
 tag = "pred")

stack <- inla.stack(stack_est, stack_pred)


# Run INLA only on monitor locations
result_est <- inla(update(formula$a, ~ . + f(j, model='iid')),
 data = inla.stack.data(stack_est, spde = spde),
 family = "gaussian",
 control.family = list(hyper = list(prec = list(initial = 20, fixed = TRUE))),
 control.predictor = list(A = inla.stack.A(stack_est),
 link = 1,
 compute = FALSE),
 control.fixed = list(expand.factor.strategy = 'inla'),
 verbose = FALSE)

# Run INLA on monitor and clinic locations
result_pred <- inla(update(formula$b, ~ . + f(j, model='iid')),
 data = inla.stack.data(stack, spde = spde),
 family = "gaussian",
 control.family = list(hyper = list(prec = list(initial = 20, fixed = TRUE))),
 control.predictor = list(A = inla.stack.A(stack),
 link = 1,
 compute = TRUE),
 control.fixed = list(expand.factor.strategy = 'inla'),
 control.mode = list(theta = result_est$mode$theta,
 restart = FALSE),
 verbose = FALSE)
