# Supplementary material for: Estimating ambient air pollutant levels in Suzhou through the SPDE approach with R-INLA
Source: Int J Hyg Environ Health. 2021 Jun;235:113766. doi: 10.1016/j.ijheh.2021.113766 (PMC8223501; doi:10.1016/j.ijheh.2021.113766)
Supplement: Multimedia component 2 [file mmc2.docx]

# Definitions of models for weather variables and pollutants

The SPDE approach to spatial modelling and the Matérn covariance function and its parameters are well described in Chapter 6 of Blangiardo and Cameletti (2015). The general form of the spatio-temporal models used in this paper is well described in Chapter 7 of Blangiardo and Cameletti (2015) and Chapter 10 of Moraga (2019).

## Weather models

For each standardised weather variable (temperature, humidity, log transformed wind speed, precipitation), $y$, we use the model

$$\begin{matrix} y_{it}\sim& \text{Normal}\left( \eta_{it}, \sigma_{e}^{2} \right) \\ \eta_{it}= & \boldsymbol{\beta}_{\mathbf{0}}\mathbf{y}\mathbf{m}\mathbf{o}\mathbf{n}\mathbf{t}\mathbf{h}_{\mathbf{t}}+\omega_{it} \end{matrix}$$

where $i$ indexes location and $t$ indexes time (months). Note that $\boldsymbol{\beta}_{\mathbf{0}}$ is a vector of intercept terms for each month of the year (January, February, …) and $\mathbf{y}\mathbf{m}\mathbf{o}\mathbf{n}\mathbf{t}\mathbf{h}_{\mathbf{t}}$ is a vector indicating the month for time $t$.

$\omega_{it}$ is the spatio-temporal random effect, with first order auto-regressive model for temporal correlation:

$$\begin{matrix} \omega_{it}= & a\omega_{i(t-1)}+\xi_{it} \\ \omega_{i1}\sim& \text{Normal}(0, \sigma^{2}/(1-a^{2})) \end{matrix}$$

$\xi_{it}$ is a zero-mean Gaussian field. It is temporally independent but spatially correlated at each time point with Matérn covariance function. $a$ is the coefficient of the autoregressive process.

Priors for parameters:

$$\begin{matrix} \boldsymbol{\beta}_{\mathbf{0}}\overset{iid}{\sim} & \text{Normal}(0, 1000) \\ 1/\sigma_{e}^{2}\sim& \text{Gamma}(1, 0.00005) \\ \text{log}\left( (1+a)/(1-a) \right)\sim& \text{Normal}(0,{}^{1}/_{0.15}) \end{matrix}$$

For the parameters of the Matérn covariance, penalised complexity (PC) priors are used so that $P(r<10)=0.5$ and $P(\sigma>1)=0.5$, where $r$ is the range and $\sigma$ the standard deviation of the field.

For $\text{Gamma}(a, b)$, $a$ is the shape parameter and $b$ is the inverse scale parameter, so that the distribution has mean $a/b$ and variance $a/b^{2}$.

## Pollutant models

For each log transformed pollutant, $y$, we use the model

$$\begin{matrix} y_{it}\sim& \text{Normal}(\eta_{it},\sigma_{e}^{2}) \\ \eta_{it}= & \boldsymbol{\beta}_{\mathbf{0}}\mathbf{y}\mathbf{m}\mathbf{o}\mathbf{n}\mathbf{t}\mathbf{h}_{\mathbf{t}}+\beta_{1}\text{month}_{t}+ \\ & \beta_{2}\text{long}_{i}+\beta_{3}(\text{long}^{2})_{i}+\beta_{4}\text{lat}_{i}+\beta_{5}(\text{lat}^{2})_{i}+ \\ & \beta_{6}\text{elevation}_{i}+\beta_{7}\text{distroad}_{i}+\beta_{8}\text{distmway}_{i}+\beta_{9}\text{lengthroad}_{i}+\beta_{10}\text{urban}_{i}+ \\ & \beta_{11}\text{temp}_{it}+\beta_{12}\text{windspeed}_{it}+\beta_{13}\text{humidity}_{it}+\beta_{14}\text{precipitation}_{it}+ \\ & \omega_{it} \end{matrix}$$

where $i$ indexes location and $t$ indexes time (months). Note that $\boldsymbol{\beta}_{\mathbf{0}}$ is a vector of intercept terms for each month of the year and $\mathbf{y}\mathbf{m}\mathbf{o}\mathbf{n}\mathbf{t}\mathbf{h}_{\mathbf{t}}$ is a vector of indicator variables for month of the year. month is the number of months from January 2013, so that $\beta_{1}$ is a linear time trend.

long and lat are standardised longitude and latitude coordinates.

elevation, distroad, distmway and lengthroad are standardised variables for elevation, distance to nearest major road, distance to nearest motorway, and total length of major roads and motorways in a 1km radius. urban is a binary variable for land use.

$\omega_{it}$ is the spatio-temporal random effect, with first order auto-regressive model for temporal correlation:

$$\begin{matrix} \omega_{it}= & a\omega_{i(t-1)}+\xi_{it} \\ \omega_{i1}\sim& \text{Normal}(0,\sigma^{2}/(1-a^{2})) \end{matrix}$$

$\xi_{it}$ is a zero-mean Gaussian field. It is temporally independent but spatially correlated at each time point with Matérn covariance function. $a$ is the coefficient of the autoregressive process.

### Fixed values for weather variables

Two of the four approaches to include weather covariates in the pollutant models used fixed values. In these models, temp, windspeed, humidity, and precipitation are observed covariates, and $\beta_{11},\beta_{12},\beta_{13},\beta_{14}$ are fixed effect parameters.

### Error model for weather variables

In models where an error model is used to include predicted values of the weather covariates, a Berkson error model is used. For example, for temperature:

$$\begin{matrix} \text{temp}_{it}=\text{fitted-temp}_{it}+u_{it} \\ u_{it}\sim\text{Normal}(0,\sigma_{\text{temp},it}^{2}) \end{matrix}$$

where $\text{fitted-temp}_{it}$ is the mean of the posterior predictive distribution (at location $i$ and time $t$) and $\sigma_{\text{temp},it}^{2}$ is the variance of the posterior predictive distribution (at location $i$ and time $t$) from the associated weather model. Note that $\sigma_{\text{temp},it}^{2}$ are fixed values, not parameters.

### Priors for parameters:

Intercept and fixed effect parameters:

$$\boldsymbol{\beta}_{\mathbf{0}},\beta_{1},\ldots,\beta_{10}\overset{iid}{\sim}\text{Normal}(0, 1000)$$

In models using fixed weather covariates:

$$\beta_{11},\beta_{12},\beta_{13},\beta_{14},\overset{iid}{\sim}\text{Normal}(0, 1000)$$

In models using Berkson error models for weather covariates:

$$\beta_{11},\beta_{12},\beta_{13},\beta_{14},\overset{iid}{\sim}\text{Normal}(1, 1000)$$

Other parameters:

$$\begin{matrix} 1/\sigma_{e}^{2}\sim& \text{Gamma}(1, 0.00005) \\ \text{log}\left( (1+a)/(1-a) \right)\sim& \text{Normal}(0,{}^{1}/_{0.15}) \end{matrix}$$

For the parameters of the Matérn covariance, penalised complexity (PC) priors are used so that $P(r<10)=0.5$ and $P(\sigma>1)=0.5$, where $r$ is the range and $\sigma$ the standard deviation of the field.

## Pollutant models without spatial correlation

In models excluding the SPDE model, but including the temporal first order auto-regressive random effects for stations:

$$\begin{matrix} \omega_{it}= & a\omega_{i(t-1)}+\xi_{it} \\ \omega_{i1}\sim& \text{Normal}(0, \sigma^{2}/(1-a^{2})) \\ \xi_{it}\sim& \text{Normal}(0, \sigma_{s}^{2}) \end{matrix}$$

The prior for $\sigma_{s}^{2}$ is given by $(1/\sigma_{s}^{2})(1-a^{2})\sim\text{Gamma}(1, 0.00005)$.

# Bibliography

Blangiardo, M., Cameletti, M., 2015. Spatial and Spatio-temporal Bayesian Models with R - INLA. Wiley.

Moraga, P., 2019. Geospatial Health Data: Modeling and Visualization with R-INLA and Shiny. Chapman; Hall/CRC.
